# Development of a genetic system for the deep-sea psychrophilic bacterium *Pseudoalteromonas* sp. SM9913

**DOI:** 10.1186/1475-2859-13-13

**Published:** 2014-01-22

**Authors:** Zi-Chao Yu, Dian-Li Zhao, Li-Yuan Ran, Zi-Hao Mi, Zhao-Yu Wu, Xiuhua Pang, Xi-Ying Zhang, Hai-Nan Su, Mei Shi, Xiao-Yan Song, Bin-Bin Xie, Qi-Long Qin, Bai-Cheng Zhou, Xiu-Lan Chen, Yu-Zhong Zhang

**Affiliations:** 1 State Key Laboratory of Microbial Technology, Shandong University, Jinan 250100, China; 2 Marine Biotechnology Research Center, Shandong University, Jinan 250100, China

## Abstract

**Background:**

*Pseudoalteromonas* species are a group of marine gammaproteobacteria frequently found in deep-sea sediments, which may play important roles in deep-sea sediment ecosystem. Although genome sequence analysis of *Pseudoalteromonas* has revealed some specific features associated with adaptation to the extreme deep-sea environment, it is still difficult to study how *Pseudoalteromonas* adapt to the deep-sea environment due to the lack of a genetic manipulation system. The aim of this study is to develop a genetic system in the deep-sea sedimentary bacterium *Pseudoalteromonas* sp. SM9913, making it possible to perform gene mutation by homologous recombination.

**Results:**

The sensitivity of *Pseudoalteromonas* sp. SM9913 to antibiotic was investigated and the erythromycin resistance gene was chosen as the selective marker. A shuttle vector pOriT-4Em was constructed and transferred into *Pseudoalteromonas* sp. SM9913 through intergeneric conjugation with an efficiency of 1.8 × 10^-3^, which is high enough to perform the gene knockout assay. A suicide vector pMT was constructed using pOriT-4Em as the bone vector and *sacB* gene as the counterselective marker. The *epsT* gene encoding the UDP-glucose lipid carrier transferase was selected as the target gene for inactivation by in-frame deletion. The *epsT* was in-frame deleted using a two-step integration–segregation strategy after transferring the suicide vector pMT into *Pseudoalteromonas* sp. SM9913. The Δ*epsT* mutant showed approximately 73% decrease in the yield of exopolysaccharides, indicating that *epsT* is an important gene involved in the EPS production of SM9913.

**Conclusions:**

A conjugal transfer system was constructed in *Pseudoalteromonas* sp. SM9913 with a wide temperature range for selection and a high transfer efficiency, which will lay the foundation of genetic manipulation in this strain. The *epsT* gene of SM9913 was successfully deleted with no selective marker left in the chromosome of the host, which thus make it possible to knock out other genes in the same host. The construction of a gene knockout system for *Pseudoalteromonas* sp. SM9913 will contribute to the understanding of the molecular mechanism of how *Pseudoalteromonas* adapt to the deep-sea environment.

## Background

The deep-sea floor constitutes almost 60% of the earth’s surface [1], most of which is covered with fine-grained sediments [2]. The deep-sea sediments are a greatly dynamic geo- and biosphere, where no light is present, the temperature is as low as −1°C to 4°C and the pressure is up to 1,100 bar [2]. Despite these extreme conditions, various microorganisms flourish in deep-sea sediments. Approximately 13% of the total global bacteria have been found in the upper 10 cm of deep-sea sediments [3,4], which play an important role in the deep-sea ecosystem.

The extreme characteristics of the deep-sea sediments have compelled various bacteria to evolve special features to adapt to the deep-sea environment. However, the molecular mechanism of deep-sea bacteria adapted to deep-sea sediments is still largely unknown. By far, only several strains, such as *Photobacterium profundum* SS9 and *Shewanella piezotolerans* WP3, have been well studied in their adaptation to the deep sea. *Photobacterium profundum* SS9, which was isolated from the Sulu Sea in Philippines at a depth of 2.5 km [5], was found to have many special genetic features in DNA replication, fidelity and structure [6,7], as well as membrane integrity and fluidity [8-10] to adapt to the high-pressure environment by Tn5 gene replacement mutagenesis. The environmental adaptation mechanisms of *Shewanella piezotolerans* WP3, a bacterium isolated from a western Pacific Ocean sediment sample at a depth of 1914 m [11], was also studied in the genomic level [12]. Furthermore, many genes of this strain were found to be regulated by cold shock [13], and the regulation of fatty acid biosynthesis and NAP-α system in response to different temperatures and pressures were studied by gene knockout [14,15].

*Pseudoalteromonas* are widespread in various marine environments. Over 30 marine species with a large number of strains have been reported in this genus. Studies on these marine bacteria have suggested that they play important roles in marine ecosystem. However, by far in this genus, only one strain, *Pseudoalteromonas haloplanktis* TAC125 from the Antarctic seawater, has been reported to have a gene knockout genetic system [16]. *Pseudoalteromonas* strains are also frequently encountered in the analysis of deep-sea sedimentary bacterial diversity [17-19], implying that some bacteria in this genus may also play an important role in deep-sea sediment ecosystem. Some *Pseudoalteromonas* strains isolated from deep-sea sediments have been shown to be good extracellular enzyme producers or exopolysaccharide (EPS) producers [18,20-23]. It is reported that an electroporation transformation system was developed in *Pseudoalteromonas* sp. PS1M3 from deep-sea sediments [24]. Nevertheless, gene deletion has not been performed in this strain. Thus, gene knockout genetic system has not yet constructed in a deep-sea *Pseudoalteromonas* strain. Due to the lack of such genetic systems, little is yet known how *Pseudoalteromonas* adapt to the deep-sea environment at molecular level.

*Pseudoalteromonas* sp. SM9913 (hereafter called SM9913), isolated from the deep-sea sediment at 1855 m depth near the Okinawa Trough [25], is a psychrophilic strain capable of producing a great quantity of proteases [20,26,27] and EPS [21]. The genome of SM9913 has been sequenced, and some specific features implying how *Pseudoalteromonas* sp. SM9913 adapts to the deep-sea environment have been revealed in gene level [28]. However, it is still difficult to verify the predicted features by experiments due to the lack of a genetic system in SM9913. Therefore, it is necessary to develop a genetic system for the reverse genetics in SM9913 to investigate the molecular mechanism of how this bacterium adapts to the deep-sea sedimentary environment. In this study, a conjugal transfer system with high efficiency was constructed, and a gene knockout system in SM9913 was developed with the *epsT* gene encoding the initial UDP-glucose lipid carrier transferase as the target.

## Results and discussion

### Sensitivity of SM9913 to six antibiotics

The antibiotics sensitivity assay of SM9913 was performed with six antibiotics. The result showed that SM9913 was completely resistant to ampicillin, kanamycin, tetracycline and streptomycin, partly sensitive to chloromycetin and completely sensitive to erythromycin of 100 μg/ml (Table 1). This result is consistent with the information obtained from genome analyisis of SM9913. SM9913 harbors 12 multidrug-resistance genes, 4 β-lactamase genes and genes encoding AcrA/E family efflux transporter and AcrB/AcrD/AcrF family protein in its genome [28]. These genes are all involved in drug resistance [29]. Although the growth of SM9913 was not completely restrained by 50 μg/ml erythromycin, SM9913 could not survive when erythromycin of 100 μg/ml were added to the medium. Therefore, 100 μg/ml erythromycin was used for screening SM9913 mutants in this study.

**Table 1:** Sensitivity of SM9913 to different antibiotics

	**10 μg/ml**	**50 μg/ml**	**100 μg/ml**
Ampicillin	- - -	- - -	- - -
Kanamycin	- - -	- - -	- - -
Chloromycetin	- - -	+ +	+ +
Erythromycin	+	+ +	+ + +
Tetracycline	- - -	- - -	- - -
Streptomycin	- - -	- - -	- - -

+: sensitivity. -: insensitivity.

The number of + or – represent the level of sensitivity or insensitivity to antibiotics of different concentrations.

### Construction of the conjugal transfer system with high efficiency

**Figure 1:**
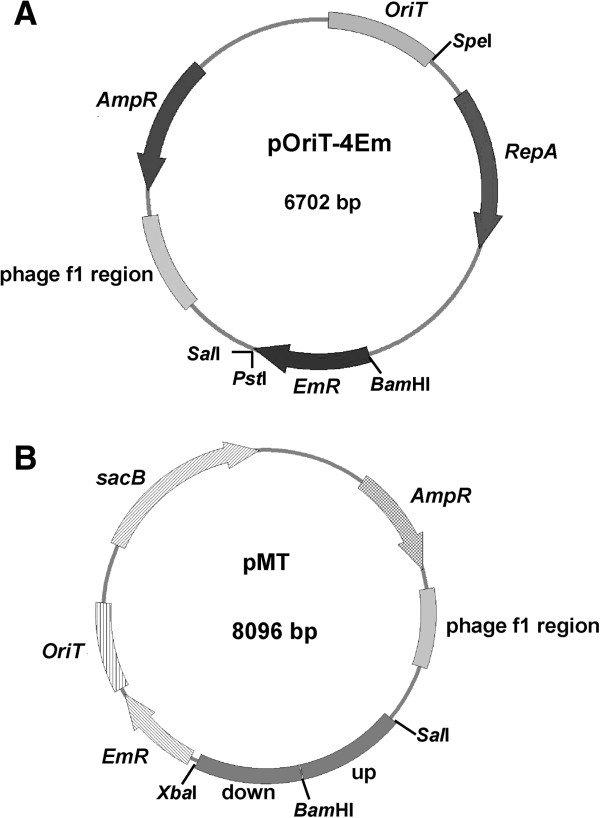
Maps of the shuttle vector pOriT-4Em (A) and the suicide vector pMT (B).

A shuttle vector, pOriT-4Em, which can replicate in both *E. coli* and SM9913, was constructed. As shown in Figure 1A, pOriT-4Em is characterized by the presence of a DNA fragment containing conjugative transfer initiation origin (*OriT*) and selective marker genes of ampicillin resistance gene (*AmpR*) and erythromycin resistance gene (*EmR*). The phage f1 region on pOriT-4Em is responsible for the replication of pOriT-4Em in *E. coli* DH5α or *E. coli* ET12567 (pUZ8002). *RepA* gene and its flanking sequences on pOriT-4Em, derived from pSM429 (a plasmid from the sea-ice bacterium *Pseudoalteromonas* sp. BSi20429 [30]), were responsible for the replication and segregational stability of pOriT-4Em in SM9913.

**Figure 2:**
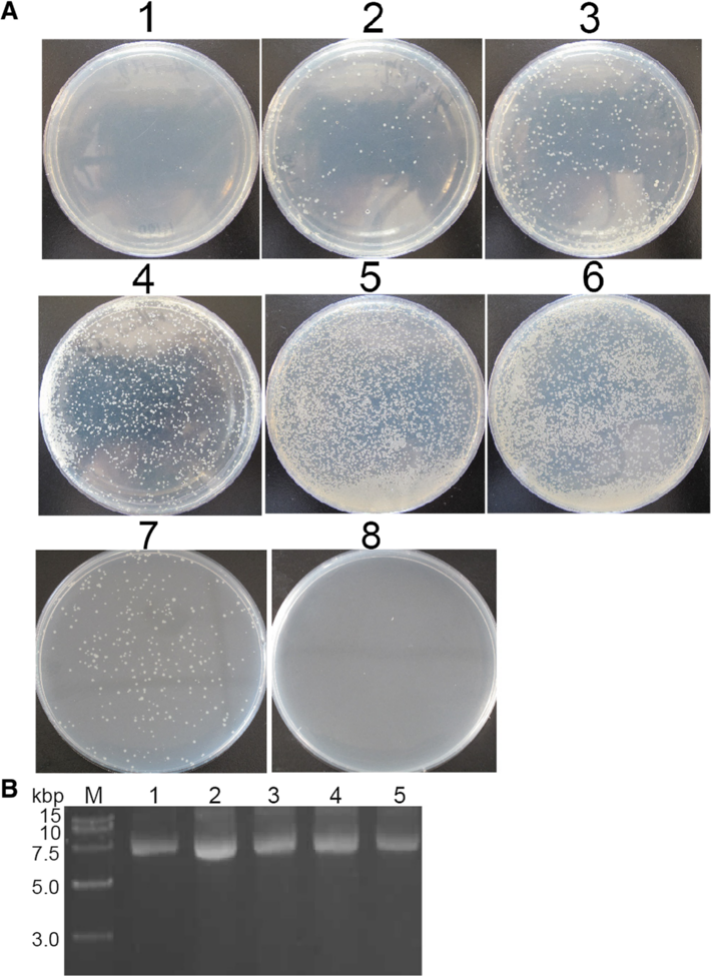
**Screening of the transconjugants on the selective plates. (A)** Photos of the transconjugants on the selective plates containing erythromycin after donor and recipient cells were mixed in different proportions. 1, donor : recipient = 1 : 100; 2, donor : recipient = 1 : 10; 3, donor : recipient = 1 : 5; 4, donor : recipient = 1 : 1; 5, donor : recipient = 10 : 1; 6, donor : recipient = 100 : 1; 7, *E. coli* ET12567 harboring pOriT-4Em-*ΔRepA* mixed with marine LB medium; 8, *E. coli* ET12567 harboring pOriT-4Em-Δ*RepA*: SM9913 = 100 : 1. In photos 1 – 6, the donor *E. coli* ET12567 harbored pOriT-4Em. **(B)** Electrophoresis analysis of the vector pOriT-4Em extracted from the transformed SM9913. Lanes: M, DNA marker; 1, pOriT-4Em extracted from *E. col*i DH5α used as the positive control. 2 – 5, pOriT-4Em extracted from SM9913.

pOriT-4Em was transferred into SM9913 by conjugal transfer using *E. coli* ET12567 (pUZ8002) as the donor, SM9913 as the recipient and erythromycin for screening. Transconjugant colonies were visible on the selective plates at 20°C after 36 – 48 h incubation. The transfer efficiency was affected by the proportion of donor cells and recipient cells (Figure 2A). The optimal proportion was donor: recipient = 100: 1. Plasmids were extracted from the transconjugants cultured in marine LB broth at 20°C overnight to exclude the false positive clones. The result showed that all tested transconjugants harbored the vector pOriT-4Em (Figure 2B). In this experiment, we found that it seemed that the donor *E. coli* ET12567 could not survive on the selective plate at 20°C after mating with SM9913 for unknown reason. To confirm this, a vector pOriT-4Em-*ΔRepA* that cannot replicate in SM9913 was constructed, and conjugation assay was performed using pOriT-4Em-*ΔRepA* as a control. Although *E. coli* ET12567 harboring pOriT-4Em-*ΔRepA* alone could grow on the selective plate at 20°C after 7 day incubation, it could not survive after mating with SM9913 (Figure 2A). This result confirmed that the donor *E. coli* ET12567 cells could not survive after the conjugation, though the reason underlying this phenomenon is not clear yet. Therefore, it can be sure that the colonies shown on the plates 1–6 in Figure 2A should be *Pseudoalteromonas* sp. SM9913 recombinant colonies. The transfer efficiency was calculated as 1.8 × 10^-3^, which is high enough to perform the gene knockout assay.

### Generation of the *epsT* mutant strain

As shown in Figure 1B, the suicide vector pMT was constructed using pOriT-4Em as the bone vector by eliminating *RepA* and its flanking sequences, inserting fragments (up and down) that flanks the *epsT* gene, and adding counter selective marker *sacB* gene. *sacB* encodes levansucrase, whose activity will not permit the host to survive in sucrose-containing medium.

**Figure 3:**
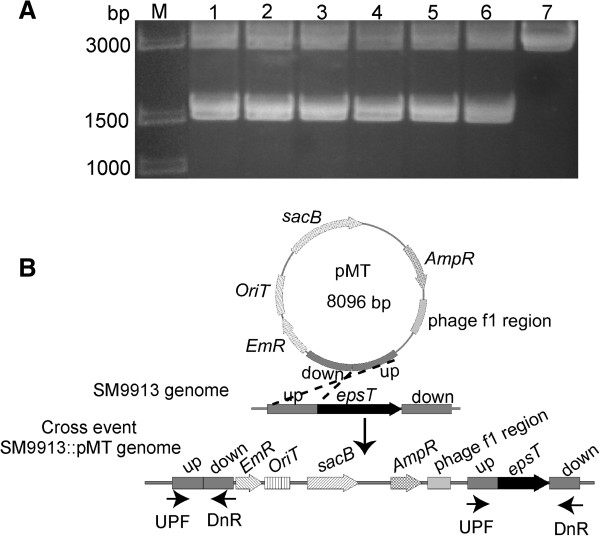
**Verification and schematic representation of the first recombination. (A)** Electrophoresis analysis of the PCR products to confirm the mutants in which the first homologous recombination making pMT insert into the chromosome of SM9913 occurred. Lanes: M, DNA marker; 1 – 6, pMT inserted mutants; 7, wild SM9913. **(B)** Schematic representation of the first recombination event and positions of the primers for mutation verification. UPF and DnR were the primers used for mutation verification.

The suicide vector pMT was mobilized into SM9913 by intergeneric conjugation. Cells were plated on marine LB solid medium containing erythromycin to screen the clones in which a single crossing over event occurred. In the first homologous recombination, pMT was inserted into the chromosome of SM9913. Since pMT contains two homologous fragments, two different crossing over events may occur depending on which fragment underwent recombination. No matter which crossing over event occurred, each homologous fragment always had two copies in the genome. The *epsT* gene is flanking by a pair of homologous fragments and the other pair of the homologous fragments was arranged like that in pMT (Figure 3B). So primers UPF/DnR were used to confirm plasmid insertion. Two DNA fragments of different molecular weights were amplified from the positive clones by PCR, as expected (Figure 3A), indicating that a single crossing over event occurred in these clones.

**Figure 4:**
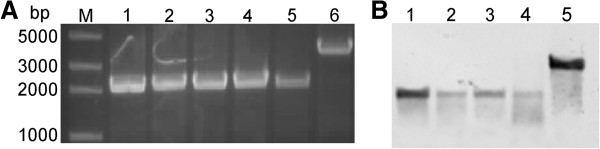
**Confirmation of the mutants that underwent the second homologous recombination. (A)** Electrophoresis analysis of the PCR products to confirm the cross event. Lanes: M, DNA marker; 1 – 5, *epsT* deleted mutants; 6, wild type of SM9913. **(B)** Southern blot hybridization of the mutants and wild SM9913. Lanes: 1 – 4, the deleted mutants; 5, wild type of SM9913.

The levansucrase encoded by *sacB* gene catalyzes hydrolysis of sucrose and synthesis of levans, which are high-molecular-weight fructose polymers [31]. The expression of *sacB* in some gram-negative bacteria is fatal in the presence of sucrose [32], making it a good candidate for counter selection. So the second homologous recombination in SM9913 was performed on the marine LB solid medium containing 30% sucrose. The mutants were verified by PCR analysis using primers TyzF/TyzR flanking the *epsT* and by Southern blot hybridization (Figure 4A,B). The results showed that pMT had excised from the target locus in the chromosome of SM9913. Moreover, depending on the site of recombination, back mutants and deletion mutants could be both generated, although back mutants were not selected in this study.

### Effect of *epsT* knockout on the EPS production of SM9913

The *epsT* gene of SM9913 encodes the UDP-glucose lipid carrier transferase, which may play an important role in the EPS synthesis in SM9913. To determine the effect of *epsT* knockout on the EPS production of SM9913, wild-type SM9913 and the *ΔepsT* mutant were cultured in marine LB broth, and the EPS yields were measured. The EPS produced by wild SM9913 in marine LB broth were 96 mg/l, and those produced by the *ΔepsT* mutant were only 26 mg/l. Therefore, knockout of *espT* severely affected the EPS production of SM9913, indicating that *epsT* is an important gene involved in the EPS biosyntheis of SM9913. The residual level of EPS in the *ΔepsT* mutant suggests that there may be either other gene(s) homologous to *epsT* or other pathway involved in EPS biosynthesis in SM9913.

In genus *Pseudoalteromonas,* a gene knockout genetic system has been developed in psychrophilic *Pseudoalteromonas haloplanktis* TAC125 from the Antarctic seawater [16]. In this study, a gene knockout genetic system was successfully developed in the deep-sea psychrophilic strain *Pseudoalteromonas* sp. SM9913. In the genetic system of *Pseudoalteromonas haloplanktis* TAC125, 4°C was used to select the transconjugants to avoid the growth of the donor cells [16,33]. In contrast, the transconjugants of SM9913 could be obtained at a wide range of temperatures below 20°C without the growth of the donor cells. This can save time because SM9913 grows much faster at its optimal temperature 15-20°C than at 4°C. Moreover, a wide temperature range for transconjugant selection would be helpful for studying the function of genes related to cold-adaptation of psychrophilic bacteria. For some key genes associated with bacterial cold-adaptation, the disruption of them may make bacterial cells difficult to grow at temperature as low as 4°C, which would disable the selection of transconjugants that have undergone first or double cross-over. In SM9913, this situation can be avoided by selecting the transconjugants at 20°C.

## Conclusions

*Pseudoalteromonas* sp. SM9913 is a model strain to study the mechanism of how *Pseudoalteromonas* adapt to extreme deep-sea environment. In this study, the antibiotic sensitivity of *Pseudoalteromonas* sp. SM9913 was investigated. Erythromycin was selected for the screening of the transconjugants and the mutants that have underwent the first homologous recombination. A conjugal transfer system was constructed in SM9913 with a wide range of selection temperatures below 20°C and a high transfer efficiency of 1.8 × 10^-3^, which is high enough for the genetic manipulation such as heterologous expression and gene knockout in *Pseudoalteromonas* strains. The *epsT* gene of SM9913 was successfully deleted with a two-step homologous recombination without introducing any selective marker, making it possible to knock out other genes in the same host. Thus, a method for gene knockout in *Pseudoalteromonas* sp. SM9913 was successfully constructed, which will facilitate the functional analysis of genes associated with the deep-sea environment adaption of *Pseudoalteromonas*.

## Materials and methods

### Plasmids, bacterial strains and growth conditions

The plasmids and bacterial strains used in this study were described in Table 2. SM9913 was grown at 15-20°C in a marine LB broth (10 g peptone, 5 g yeast extract, 1 l artificial seawater, pH 7.5). *Escherichia coli* DH5α used as the host for gene cloning was grown at 37°C in LB medium [34]. *E. coli* ET12567 harboring plasmid pUZ8002, a derivative of RK2 plasmid with a mutation in *oriT*, was used as the donor in intergeneric conjugation experiments, which was cultured at 37°C in LB medium containing chloromycetin (25 μg/ml) and kanamycin (100 μg/ml) because pUZ8002 contains these two resistance genes.

**Table 2:** Strains and plasmids used in this study

**Strains or plamids**	**Genotype or markers; characteristics and uses**	**Source or reference**
Strains		
*Escherichia coli* DH5α	*supE* 44, △*lac*U169 (θ80*lacZ*△*M15*) *endA1, recA1, hsdR17*, *thi-1* l − *gyrA96, relA1*; gene cloning	Transgen, China
*Escherichia coli* ET12567	*dam, dcm, hsdM, hsdS, hsdR, cat, tet*; donor for the conjugal transfer	[35]
*Pseudoalteromonas* sp. SM9913	Wild type	This work
Plasmids		
pUZ8002	*tra, neo,* RP4; a derivative of RK2 with a mutation in *oriT*	[36]
pKNG101	R6K-derived suicide plasmid containing *Str* and *sacB*; template for *oriT* and *sacB* amplification	[37]
pSM429	Cryptic plasmid of *Pseudoalteromonas* sp. Bsi20429; template for *RepA*	[30]
pMG36e	Em^R^, pWV01 origin, expression vector for *L. lactis*; template for *EmR* amplification	[38]

### Antibiotics sensitivity assay

Ampicillin, kanamycin, chloromycetin, erythromycin, tetracycline and streptomycin were used for the antibiotics sensitivity assay of SM9913. SM9913 was grown in marine LB broth at 20°C to late exponential phase, and then 1% of the culture was inoculated to marine LB broth containing one of the six antibiotics in the final concentration of 10 μg/ml, 50 μg/ml or 100 μg/ml. The culture containing no antibiotic was used as the control. The cultures were incubated at 20°C with shaking for 48 h, and then were collected to determine their absorbance of OD_600_ using a V550 spectrophotometer (Jasco, Japan).

### Construction of the shuttle vector pOriT-4Em

The *OriT* gene, the conjugative transfer initiation origin, was cloned into pGEM-T Easy vector (Promega, USA) after amplified from pKNG101 using primers OriTF/OriTR (Table 3). Subsequently, a DNA fragment containing the replication protein gene *RepA* and its flanking sequences that are responsible for stable segregation of the plasmid in SM9913 [30] and erythromycin resistance gene *EmR* containing its promoter were inserted into the derivative vector at the *Spe*I and *Pst*I sites. The *RepA* fragment was amplified from pSM429 with the primers ZF/ZR [30]. The *EmR* gene containing its promoter was amplified from pMG36e using the primers EmCFB/EmC. Primers ZR and EmCFB were completely reverse complement, and the two DNA fragments were spliced by overlap PCR with introducing a *Bam*HI site between them. The constructed shuttle vector, which can replicate in both *E. coli* and SM9913, was named as pOriT-4Em.

**Table 3:** Primers used in this article

**Primer**	**Sequence (5′-3′)**	**Description**	**Expected size (bp)**
OriTF	GCCAGCTCGTCGGTGTAGC	OriT	700
OriTR	CAACAACGTTGCCCGGATCG		
ZF	TGCACTGCAGCAAGACACTGTGAAGGC	RepA and its flanking sequences	2100
ZR	GATTCATTATAACCACGGATCCCTGCCTTTAAGATTTG		
EmCFB	CAAATCTTAAAGGCAGGGATCCGTGGTTATAATGAATC	EmR in pOriT-4Em	825
EmC	GGACTAGTGTTAAGGGATGCAGTTTATG		
UPF	ACGCGTCGACGTATGGTGCTGCTGATAACAGC	Upstream homologous fragment of *epsT*	834
UPR	CGCGGATCCCAGTGTGAGTAGCACCTCAC		
DnF	CGGGATCCGTGAAAATGCGTACTAA	Downstream homologous fragment of *epsT*	830
DnR	CATTATAACCACTCTAGACCACTAAAGTTATCG		
EmF	CGATAACTTTAGTGGTCTAGAGTGGTTATAATG	*EmR* in pMT	825
EmR	CTAGCTAGCGTTAAGGGATGCAGTTTATG		
sacbF	CATGCCATGGCACATATACCTGCCGTTCAC	*sacB*	1900
sacBR	CCGGGCCCAATGCCAATAGGATATCGGC		
TyzF	GACGATGAATGGAGTGGTAAGATAG	Confirmation of the second recombination	3400^a^
TyzR	CTCCAATCATGCTGCCATGTTGC		2000^b^

^a^the size of the wild type.

^b^the size of the deleted mutant.

### Construction of the suicide vector for *epsT* gene knockout in SM9913

Construction of the suicide vector used for gene knockout was based on the shuttle vector pOriT-4Em. An 834 bp upstream homologous fragment was amplified from the upstream of SM9913 *epsT* gene using primers UPF/UPR and inserted into the *Sal*I and *Bam*HI sites in pOriT-4Em to replace the *EmR* fragment. A downstream homologous fragment was obtained by PCR from the downstream of SM9913 *epsT* gene using primers DnF/DnR. *EmR* gene with its promoter used as the selective marker for the first homologous recombination was amplified from pMG36e with the primers EmF/EmR. Primers DnR and EmF was reverse complement with introducing an *Xba*I site between the downstream homologous fragment and the *EmR* expression cassette. These two DNA fragments were fused by overlap PCR and *Bam*HI and *Nhe*I sites were introduced at the 5′ and 3′ ends, respectively. The overlapped PCR product was cloned into pOriT-4EM to replace the *RepA* and its flanking sequences. Finally, *sacB* gene containing its promoter used as the counter selective marker for the second homologous recombination was amplified from pKNG101 using primers sacBF/sacBR and inserted into the vector at the *Nco*I and *Apa*I sites. The constructed suicide vector for *epsT* gene knockout was named as pMT.

### Construction of conjugal transfer system using pOriT-4Em

The shuttle vector pOriT-4Em was transferred from *E. coli* ET12567 (pUZ8002) cells (donor strain) to SM9913 (recipient strain) by interspecific conjugation. The donor cells were grown to logarithmic phase of OD_600_ ≈ 0.6 in LB broth containing ampicillin (100 μg/ml), chloromycetin (25 μg/ml) and kanamycin (100 μg/ml) and recipient strains were grown to logarithmic phase of OD_600_ ≈ 0.6 in marine LB broth. The cells were washed twice using LB broth for the donor and marine LB broth for the recipient, and then were mixed under different proportions (donor : recipient =1 : 100, 1 : 10, 1 : 5, 1 : 1, 10 : 1 and 100 : 1). The mixed cells were spread onto a sterile 0.45 μm-pore-size membrane laid on a marine LB plate, which was then incubated at 20°C for mating. After 24 h, the cells were resuspended in 1 ml marine LB medium and spread onto marine LB plates containing 100 μg/ml erythromycin. The plates were incubated at 20°C for 36–48 h until the transconjugant colonies were visible. The transconjugants were inoculated into 5 ml marine LB broth containing 100 μg/ml erythromycin. After the cultures were shaken at 20°C overnight, plasmids were extracted using the High-purity Plasmid DNA Mini-preparation Kit (Bioteke, China).

To confirm that *E. coli* ET12567 could not survive on the selective plate at 20°C after mating with SM9913, the *RepA* gene in pOriT-4Em was disrupted by digestion with *Nhe*I and *Xba*I, and the derivative vector was named pOriT-4Em-*ΔRepA*, which could not replicate in SM9913*. E. coli* ET12567 harboring pOriT-4Em-*ΔRepA* was mixed with SM9913 under the proportion of 100 : 1. After mating at 20°C, the cells were spread onto marine LB plates containing 100 μg/ml erythromycin, which were then incubated at 20°C for at least 7 days. In the meantime, a control experiment was performed, in which *E. coli* ET12567 harboring pOriT-4Em-*ΔRepA* was mixed with marine LB medium and then spread onto the same selective plate, which was also incubated at 20°C for about 7 days until the colonies were visible.

The transfer efficiency was defined as the amount of transconjugants/the amount of donor cells in the mating assay.

### Construction of the *ΔepsT* mutant strain

The suicide vector pMT was mobilized into SM9913 by interspecific conjugation. After mating, the cells were spread onto marine LB plates containing 100 μg/ml erythromycin to screen the clones in which a single recombination of pMT inserting into SM9913 genome occurred. The colonies were inoculated into 5 ml marine LB broth and shaken for 24 h, and then the pMT insertion mutants were verified by PCR using primers UPF/DnR after genomic DNA was extracted with the Bacterial DNA Extraction Kit (Bioteke, China). The mutants were then grown at 20°C with shaking in liquid marine LB broth containing no antibiotic, and the culture of late logarithmic phase was inoculated into 5 ml marine LB broth containing no antibiotic and grown at 20°C with shaking until OD_600_ was 0.6. To select the mutants in which the second recombination occurred, the culture was serially diluted, spread onto marine LB plates containing 30% sucrose, and grown at 20°C until colonies appeared. After the colonies were inoculated into 5 ml marine LB broth and shaken for 24 h, the genomic DNA of the mutants was extracted and PCR was performed to verify the recombination event to be back mutation or deletion mutation. The deletion mutation was then tested by southern blot hybridization.

### Southern blot hybridization

Genomic DNA of the mutants was extracted with Bacterial DNA Extraction Kit and treated with the restriction endonuclease *Hin*dIII. The digested DNA was separated on 0.8% agarose gel by electrophoresis, and transferred to positively-charged nylon membrane (GE, UK) by capillary action. The probe containing part of *epsT* gene and its downstream was amplified from SM9913 genome DNA with primers EpsTF/DnR and labeled with digoxigenin-UTP. Detection of target DNA was performed using the DIG High Prime DNA Labeling and Detection kit (Roche, Switzerland) according to the manufacturer’s instructions.

### Fermentation and yield determination of the EPS of SM9913 and its mutant

The EPS yields of SM9913 and its *ΔepsT* mutant in marine LB broth were determined. SM9913 and the mutant were grown in the medium at 15°C for 72 h with shaking. The cultures were then centrifuged (10,000 rpm, 10 min) and the supernatants were dialyzed in deionized water. The concentration of the EPS in the supernatants was determined using the phenol-sulfuric acid method as previously reported with glucose as standard [39].

## Competing interests

The authors declare that they have no competing interests.

## Authors’ contributions

XC and YZ designed the project; ZY, DZ, LR and ZM performed the research; ZY, HS, QQ, BZ and YZ analyzed the data; ZY and XC wrote the paper; XP, BX, MS, XS and XZ critically reviewed the paper. All authors approved the final manuscript.
